# Out of the burrow and into the nest: Functional anatomy of three life history stages of *Ozaena lemoulti* (Coleoptera: Carabidae) reveals an obligate life with ants

**DOI:** 10.1371/journal.pone.0209790

**Published:** 2019-01-16

**Authors:** Wendy Moore, Andrea Di Giulio

**Affiliations:** 1 Department of Entomology, University of Arizona, Tucson, AZ, United States of America; 2 Department of Science, University of Roma Tre, Rome, Italy; University of Mississippi, UNITED STATES

## Abstract

The carabid subfamily Paussinae contains many species known to be obligately associated with ants during at least one stage of their life history. Myrmecophilous larvae have been documented for members of the tribe Paussini as well as several genera in the tribes Ozaenini, including *Physea* and *Eustra*. Here we describe the first instar larva of *Ozaena lemoulti*, and find it to be the most highly modified ozaenine larva that we have examined to date. Many structures of the larva suggest that it is a myrmecophile. Unlike all other described ozaenine larvae, which live in burrows that they construct and seal with their terminal disk, the completely unique larval morphology suggests *Ozaena* has adapted to living without the protection of a burrow and therefore must have a completely different feeding strategy than the typical ambush strategy of burrow dwelling larvae. We hypothesize that *Ozaena* larvae live in association with ants and use their long legs for running within the nest, and modifications of the mouthparts suggest the larva feeds on soft lightly sclerotized prey, such as ant brood. Our findings support an earlier hypothesis that *Ozaena* is mymecophilous during the adult stage. Comparisons of the functional anatomy of the eggs, larvae and adult between *Ozaena lemoulti* and the closely related, non-myrmecophilous general arthropod predator, *Goniotropis kuntzeni*, provide complementary, yet independent, evidence suggestive of this shift in lifestyle. We also examine and molecularly identify gut contents, providing direct evidence that adult *Ozaena* exclusively eat *Camponotus* ants. We conclude that *Ozaena* represents an independent shift to adopting a life of myrmecophily among beetles classified within the carabid subfamily Paussinae and document the morphological changes at each life history stage associated with the shift to a nest parasite lifestyle.

## Introduction

Many non-ant species have evolved adaptations to avoid ant attacks in order to exploit the rich, well-protected resources of ant nests. Among insects, beetles have most frequently transitioned from being free-living to being either partially or completely dependent on ant colonies for their survival [1]. Such transitions require many adaptations to this extreme lifestyle and in the case of holometabolous insects, such as beetles, different adaptations are required at each life history stage.

Many distant relatives within the predatory ground beetle family Carabidae live in ant nests either as larvae, as adults or both, including members of the tribes Anthiini, Graphipterini, Siagonini, Pseudomorphini, Orthogoniini, Ozaenini, and Paussini [2–8]

Species known to be most tightly integrated into the social structure of the ant society are classified in the genus *Paussus* (tribe Paussini). Commonly known as ant nest beetles, *Paussus* species have a remarkable array of morphological, chemical and acoustical adaptations for living inside ant nests [2, 5, 7, 9–16]. Adult *Paussus* are actively brought into the nests by worker ants [13]. Once inside, the adults interact directly with the queen without eliciting any reaction from the workers [13]. They have adopted a liquid diet and feed by using their sharp mandibles to pierce and suck fluids from the brood and the abdomen of the workers [14]. Female *Paussus* lay their eggs inside their host ant’s nest and the beetle larvae develop alongside the ant brood [14, 15]. These larvae are highly modified, rendering them nearly immobile. The components of their terminal disk are appressed and the discal surface is covered with modified structures that presumably help spread substances attractive to the ants [14–21]. The trochanter, femur, tibia and tarsus of their legs are fused [14, 21], and they become physogastric after the first instar [15, 21]. These nearly immobile larvae feed by sucking the juices from their neighboring ant brood, and they also solicit trophallaxis from worker ants. Third instar larvae dig burrows in the soil of the ant nest to pupate [21] (Di Giulio, pers. observations of *P*. *thomsonii*). Not surprisingly, *Paussus* species are among the most integrated myrmecophiles documented to date. They are completely dependent upon their ant hosts during their egg, larval and adult life history stages and the safety of the nest for protection during development.

**Fig 1:**
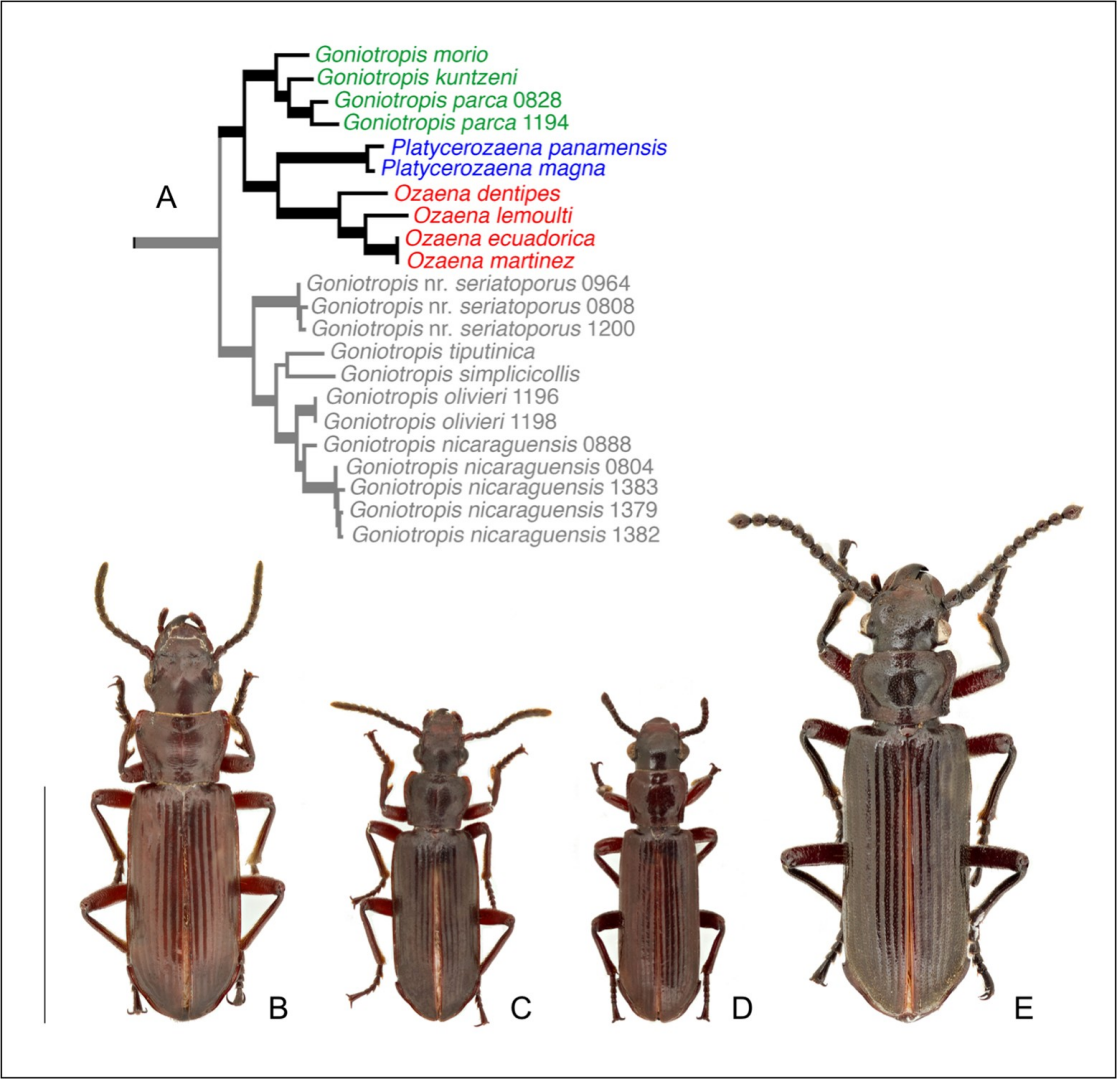
Phylogeny and dorsal habitus of adults belonging to the *Ozaena* group. (a) Bayesian consensus tree for the 28S rDNA + COI dataset of Western Hemisphere Ozaenini (modified from Moore 2008). (b) *Goniotropis kuntzeni*. (c) *Goniotropis parca*. (d) *Platycerozaena panamensis*. (e) *Ozaena lemoulti*.

Unlike their morphologically bizarre relatives in the tribe Paussini, adults and larvae in the tribes Metriini and Ozaenini resemble other free-living carabids in that they lack the morphological modifications typically associated with myrmecophily (e.g. antennomere reduction, leg modifications, cuticular grasping notches, trichomes) [1] (for example, see Fig 1B–1E). Adults are free-living general arthropod predators and while some species are found in association with (and occasionally eat) ants, they are not known to be integrated into the social life of the ant colonies. Like most carabid predators, adults use their large mandibles to crush the cuticle of their prey, which they consume in large solid pieces [22] (Moore pers. observation of *Mystropomus*, *Sphaerostylus*, *Pseudozaena*, *Pachyteles*, *Itamus*). But their larvae have a most remarkable feeding strategy. The highly mobile larvae use their mandibles and legs to construct burrows in moist wood or sandy soil, and they use the moveable components of their terminal disk like a trap door to ambush prey [23–26], http://tolweb.org/onlinecontributors/app?service=external/ViewImageData&sp=24031. Once the larva senses the presence of its prey with specialized mechanoreceptors on the terminal disk, it quickly folds the disk and extends its hyperprognathous head over its back to grasp the struggling prey with sharp mouthparts and then pulls the prey into its burrow to consume it. The burrows and the operculate terminal disks provide the otherwise vulnerable larvae with the protection they need to safely consume mobile prey. Even the few ozaenine larvae that have been collected within ant nests (*Physea* Brullé, *Eustra* Schmidt-Göbel) are thought to feed this way, ambushing ants as well as other mobile arthropods that are attracted to the rich resources of ant nests [27–29].

Here, with the first description of an *Ozaena* larva, we provide the first known exception to this pattern. *Ozaena* adults are large, parallel-sided beetles common in the New World tropics and ranging from southern Arizona to northern Argentina [30–31]. Comparative anatomy between *Ozaena* and its close relative *Goniotropis* (a typical ozaenine burrow trapper) reveals that *Ozaena* larvae have abandoned burrows to become highly mobile myrmecophiles. This represents an entirely new strategy for living with ants and an independent shift to myrmecophily within the Paussinae. Such shifts, from facultative associates to obligate associates of ants, naturally require adaptations in other life history stages, including the egg [32] and adult [1]. Based on several modifications of adult structure, George Ball and Scott McCleve predicted that species in *Ozaena* may be associated with ants [31]. Building upon their hypothesis, and our assessment of larval anatomy, we also compared *Ozaena* and *Goniotropis* egg and adult antennal morphologies. Finally, we examined and sequenced mitochondrial and ribosomal genes from adult gut contents, to further explore the hypothesis that *Ozaena* is myrmecophilous and specialized for ant predation.

## Material and methods

### Collecting notes

Adults of *Ozaena lemoulti* Bänninger, *G*. *kuntzeni*, and *G*. *parca* were collected in Walker Canyon, in the Parajito Mountains just north of the US-Mexico border in Arizona (Santa Cruz County, 31°22.819'N 111°03.994'W, 1214m). This is National Forest land, where no specific permissions are required for collecting insects by hand. Walker Canyon is a riparian oak woodland, with Emory oaks (*Quercus emoryi* Torrey) and Mexican blue oaks (*Quercus oblongifolia* Torrey), as well as species of mesquite and sycamore trees. During the summer rainy season, from July to September, *Goniotropis kuntzeni* (Fig 1B) are common in the canyon. At night the adults actively probe holes in the bark of Emory oak trees in search of prey. They are rarely, if ever, attracted to blacklights or mercury vapor lights and seem to be strictly associated Emory oaks as they have not been found on any other tree species in the canyon. *Goniotropis parca* (Fig 1C) are less common than its larger bodied sister species *G*. *kuntzeni* (Fig 1B) [33]. They also appear to be strictly associated with Emory oaks, however, they are also sometimes attracted to blacklights. *Ozaena lemoulti* (Fig 1E) adults are far less common. Over the past two decades, only six specimens of *Ozaena lemoutli* have been collected by WM in Arizona, three from Emory oaks, one from a sycamore tree, and two being captured at mercury vapor lights.

Over many years of collecting in Walker Canyon, one of us (WM) has noticed that ozaenines are only found associated with the largest Emory oak trees. Those old, but living, trees contain large dead portions, especially dead branches. Many of these trees are somewhat hollow in the middle of the trunk, and they have scars with holes on the bark left by fallen branches that appear to lead into the hollow center of the trees.

The large formicine ant, *Camponotus ocreatus* Emery, also frequents Emory oaks, and they are always found on the same large trees with ozaenines. We noted this pattern before [25], but explore it further here as *Camponotus ocreatus* is the most likely host ant of *O*. *lemoulti* larvae.

### Phylogenetic context

Southern Arizona populations of *Ozaena lemoulti* and *Goniotropis kuntzeni*, and *G*. *parca* represent the northernmost distribution of all Western Hemisphere Ozaenini, which is primarily a tropical group. These three species are all members of a small clade of Western Hemisphere ozaenines, which also includes *Goniotropis morio* Klug and the genus *Platycerozaena* (Fig 1D) [33].

### Material examined

#### Eggs

**Fig 2:**
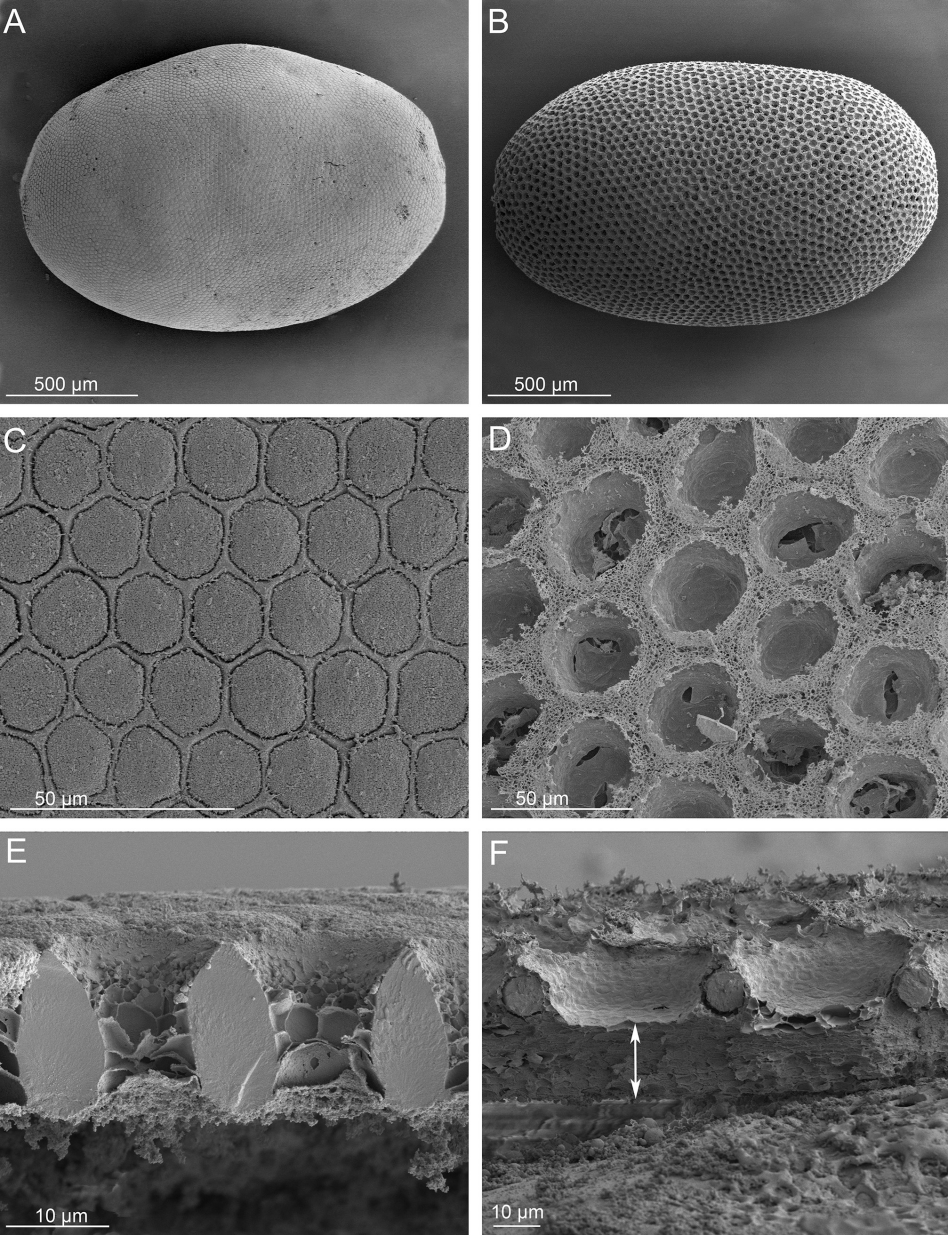
Eggs of *Ozaena lemoulti* and *Goniotropis kuntzeni*. *Ozaena lemoulti*: (a) habitus. (c) chorion surface. (e) cross section of the eggshell (note the absence of an air layer). *Goniotropis kuntzeni*: (b) habitus. (d) chorion surface. (f) cross section of the eggshell, and the thick internal air layer is denoted by a double arrow. Note the egg of *Ozaena lemoulti* is not fully mature and has spongy material on the exterior surface, the *Goniotropis kuntzeni* egg was laid in captivity.

An egg of *O*. *lemoulti* (Fig 2A, 2C and 2E) was removed from the oviducts of a dissected female which had been preserved in 100% ethanol. Five eggs of *G*. *kuntzeni* (Fig 2B, 2D and 2F) oviposited by different females from Walker Canyon were preserved in 100% ethanol.

#### Larvae

Several years ago, we reared the larvae of *Goniotropis kuntzeni*, and provided the first description of a *Goniotropis* larva [25]. For this study we reared several larvae from its sister species, *G*. *parca*, following the same methods.

**Fig 3:**
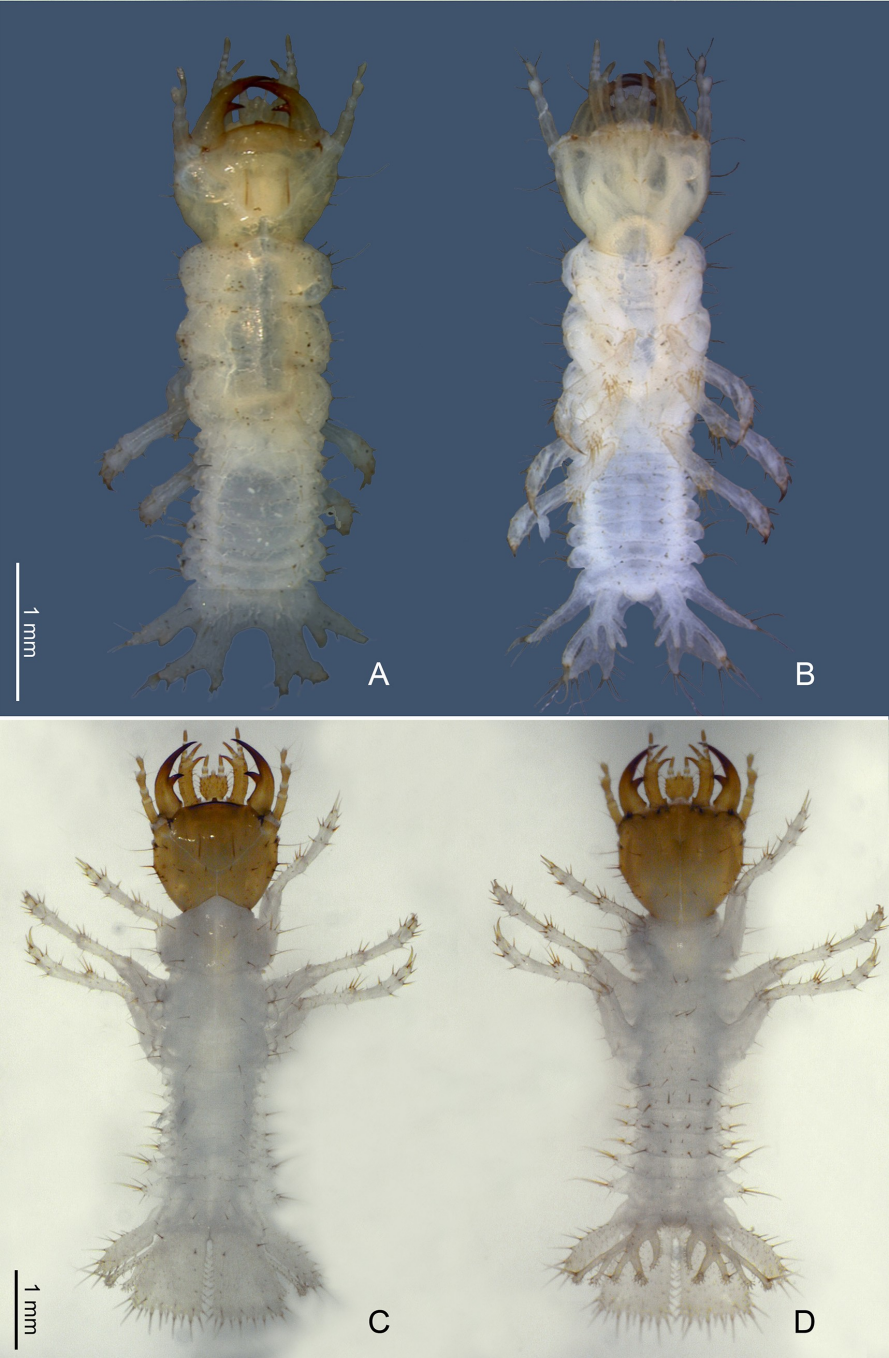
Habitus of *Ozaena lemoulti* and *Goniotropis kuntzeni* first instar larvae. *O*. *lemoulti*: (a) dorsal view. (b) ventral view. *G*. *kuntzeni*: (c) dorsal view and (d) ventral view.

We also reared a larva from a single female of *Ozaena lemoulti* (Fig 3A). The female was placed alone in a plastic chamber (5 cm X 5 cm) with peat moss (*Sphagnum* Linnaeus) dampened with distilled water. The chamber was kept at approximately 23°C near a window and thus she experienced natural lighting conditions. She seemed lethargic and many failed attempts were made to feed her with arthropod prey items that are readily accepted by *Goniotropis* species including fresh mealworm larvae (*Tenebrio* Linnaeus), fresh waxworms (Pyralidae), slices of frozen *Manduca sexta* larvae, and *Drosophila* adults. Finally, she was offered living specimens of *Camponotus ocreatus* adults which seemed to energize her. Although she was never observed feeding, several of the ant specimens disappeared from the rearing chamber and we presume that she ate them. Soon thereafter she laid an egg and she was removed from the rearing chamber. After approximately one month, a single first instar larva was found in the rearing chamber. The specimen was placed in boiling water for several seconds, then transferred to 75% EtOH. The larva and adult were deposited in the University of Arizona Insect Collection (UAIC1052874, adult; UAIC1052873, larva).

#### Adults

The right antenna of *O*. *lemoulti* and *G*. *parca* were removed from 100% ethanol preserved females from Walker Canyon. We examined the gut contents of 10 *Goniotropis* and eight *Ozaena* specimens which had been preserved in 100% EtOH. When food was present in the crop, we removed the material for molecular sequencing.

### Microscopy

All eggs, and entire first instar of *G*. *parca*, portions of the *Ozaena* larva (see below) and the adult antennae were prepared for SEM. Dried specimens were first rehydrated stepwise in a graded ethanol series to distilled water and cleaned by ultrasound for 15 seconds in a soapy water solution. All specimens were then dehydrated through a series of EtOH baths of increasing concentration (70-80-90-95-100%), critical point dried (Bal-Tec CPD 030), mounted on a stub (by using self-adhesive carbon disks), sputtered with gold (Emitech k550 sputter coater), and observed with FEI Dualbeam FIB/SEM Helios Nanolab (L.I.M.E. laboratory, University ‘Roma Tre’, Rome).

Prior to dissection, the single larva specimen of *O*. *lemoulti* was photographed and drawn using a stereomicroscope Olympus SZX16 equipped with drawing tube. The right antenna, right maxilla, right mandible, right hind leg, left dorsal and lateral plates of the terminal disk and left urogomphus were removed and prepared for scanning electron microscopy. The rest of the specimen was rehydrated, cleared in 10% KOH, transferred in hot lactic acid, dehydrated through a series of EtOH baths of increasing concentration (10-20-50-70-90-95-100%), left overnight in a clove oil bath, and mounted on a slide with Canada balsam. The slide mounted specimen was studied and illustrated by using a light microscope, Olympus BX51, equipped with drawing tube. The dissected parts of the same larva were prepared according to the procedure described above and imaged by FIB/SEM.

### Larval description

The general terminology of larval structures follows Lawrence [34]. Notation of primary setae and pores follows the system of Bousquet and Goulet [35], modified for *Metrius contractus* [17]. As some of the sensilla on the abdomen and terminal disk of *O*. *lemoulti* and *G*. *parca* are homologous to those recognized by Bousquet [17] in *Metrius contractus* (sensilla S-I to S-V), by Di Giulio et al. [36] in *Pachyteles* spp. (sensilla S-I to S-VII), and by Di Giulio and Moore [20] in *Arthropterus* sp. (sensilla S-I to S-VIII), we adopt the same nomenclature. Notation of microsculpture follows Harris [37]. An asterisk (*) following a coded seta indicates that we question the homology between the structure on the *O*. *lemoulti* and *G*. *parca* larvae.

### Molecular sequencing of adult gut contents

Total genomic DNA was extracted following the ATL protocol in the Qiagen DNeasy kit (Valenica, CA). Gene fragments from cytochrome c oxidase subunit I (COI) and 28S ribosomal DNA (28S or 28S rDNA) were amplified using the Polymerase Chain Reaction (PCR) on an Eppendorf Mastercycler Thermal Cycler with Invitrogen Platinum Taq DNA Polymerase (Carlsbad, CA).

Sequences were amplified and sequenced using the COI primers LCO1490 and HCO2198. The 28S D2/D3 region was amplified with the forward primer ls58F or ls773F (5′CACGGACCAGGGAGTCTAGCAT-3′) and the reverse primer ls1066R or ls1126R (5′TCGGAAGGAACCAGCTACTA-3′). Amplified products were cleaned, quantified, normalized and sequenced at the University of Arizona’s Genomic and Technology Core Facility using an Applied Biosystems 3730 DNA Analyzer or a 3730 XL Applied Biosystems automatic sequencer. Simultaneous contig assembly and initial base calls were performed using the Phred [38] and Phrap [39] programs as implemented in Mesquite 3.2 [40] in combination with the Chromaseq vers 1.3 package [41]. Final base calls were made after manual inspection of individual sequences in Chromaseq; universal ambiguity, IUPAC, codes were used when multiple peaks were present at individual sites. Closest published sequences to those amplified and sequenced from the food were retrieved by performing Standard Nucleotide BLAST (blastn) searches using Megablast against GenBank (NCBI).

## Results

### Comparative anatomy of eggs

The egg of *Ozaena* (Fig 2A) is 1.6 mm long and 1.1 mm wide (measured at the equatorial region), and less elliptical than the egg of *Goniotropis kuntzeni* (Fig 2B). The external surface of both eggs is covered with a grid pattern (Fig 2A and 2B). The hexagonal grids of the *Ozaena* are smaller in diameter (about 15 μm) (Fig 2C) than the more rounded grids of *Goniotropis* (about 30 μm in diameter) (Fig 2D). The *Ozaena* egg shows remnants of partially decayed follicular epithelium filling the hexagonal grids (Fig 2C). In cross section the external eggshell layer is thick (about 20 μm) and sub-oval or rhomboidal (Fig 2E) in *Ozaena*, but thin (about 10 μm) and rounded in *Goniotropis* (Fig 2F). While the internal layer of the *Goniotropis* eggshell (Fig 2F) is thick and spongy, containing air filled cavities (see also [25]) typical of other known ozaenine eggs (Type A sensu Kaupp et al. [32]), the internal layer of the *Ozaena* eggshell (Fig 2E) is thin and imperforate (similar to Type B sensu Kaupp et al. [32]).

### Comparative anatomy of larvae

**Fig 4:**
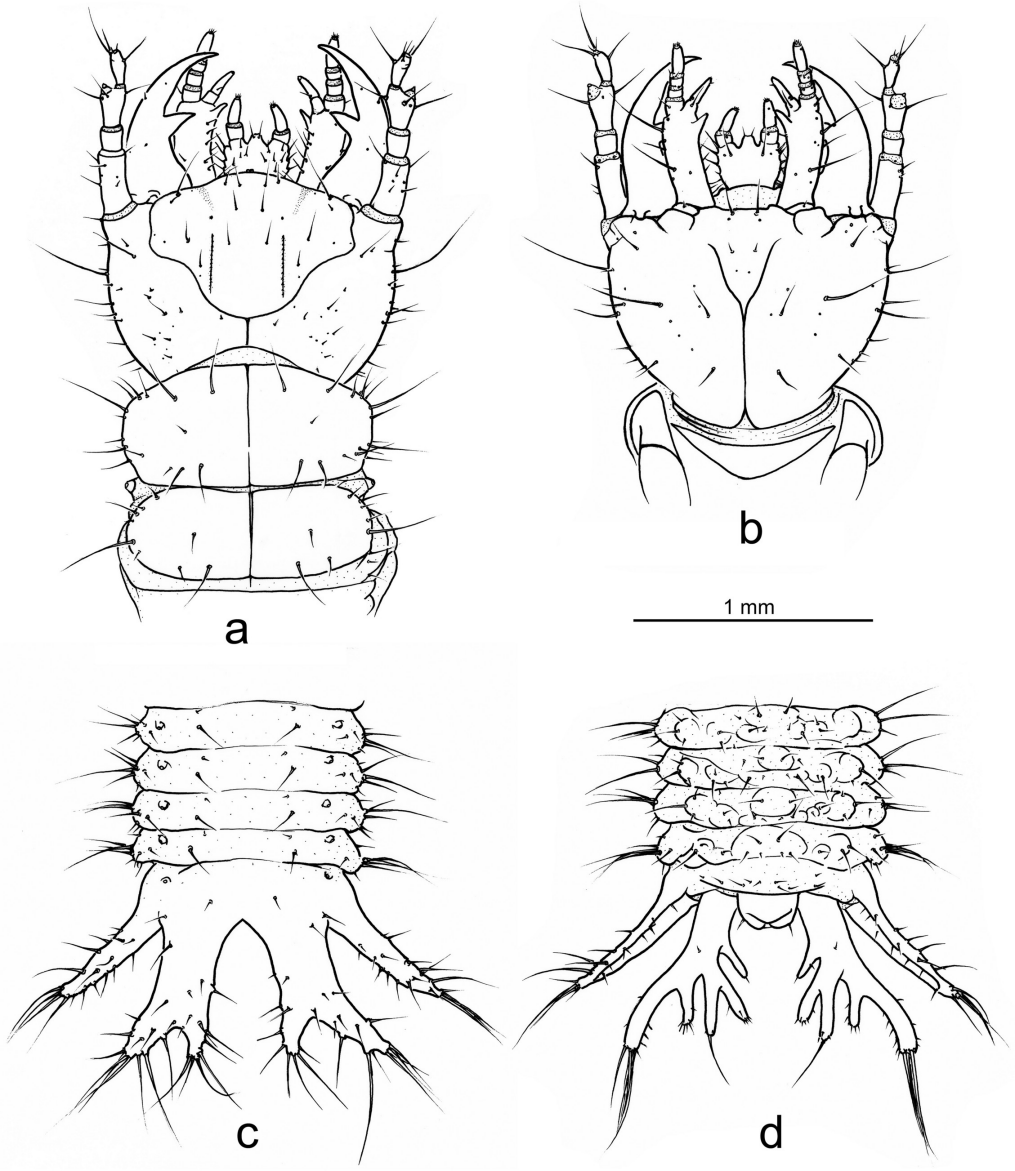
*Ozaena lemoulti* first instar larva. (a) head, pro- and mesonotum, dorsal view (b) head, ventral view. (c) apex of abdomen and folded terminal disk, dorsal view. (d) apex of abdomen and folded terminal disk, ventral view.

**Fig 5:**
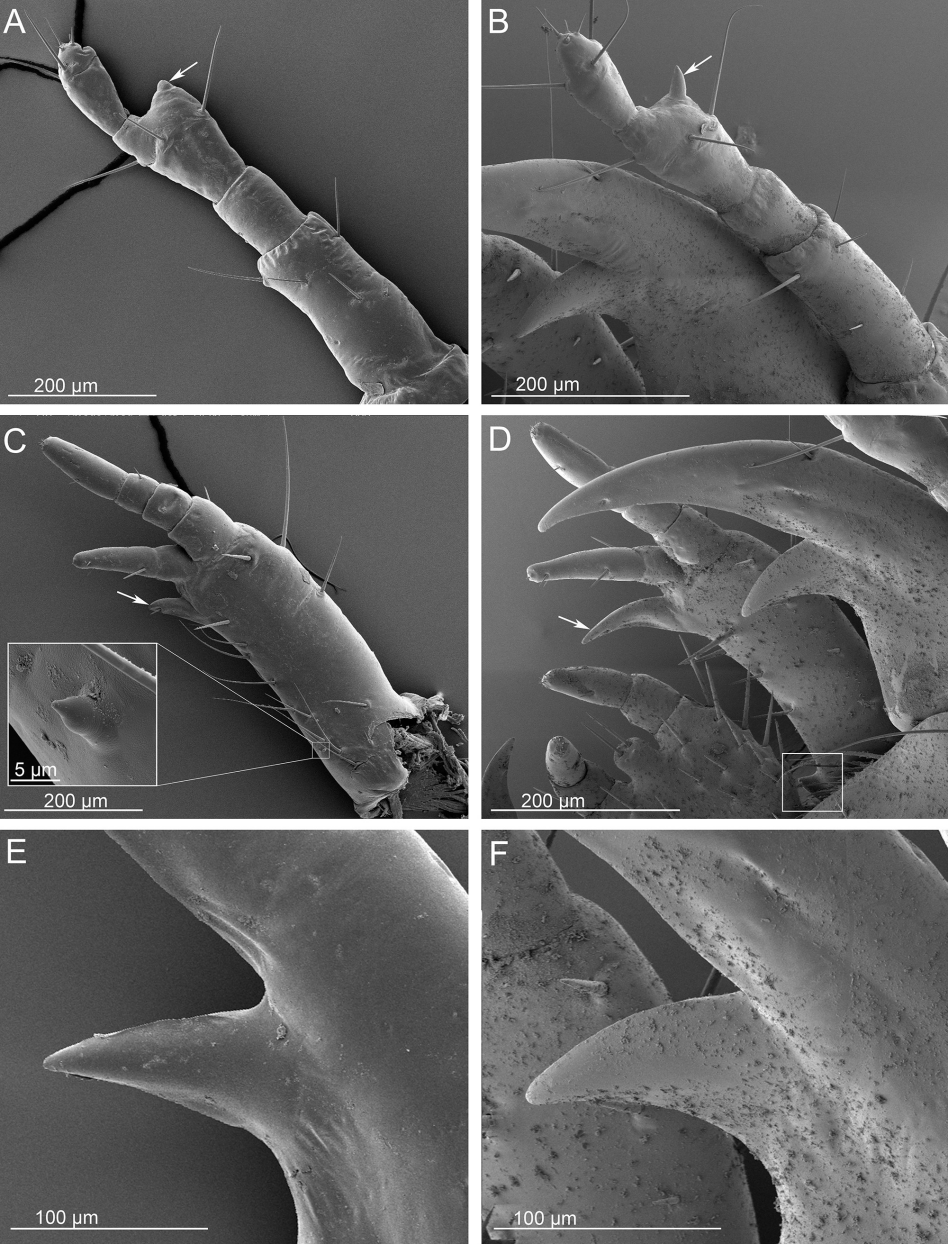
Head appendages of *Ozaena lemoulti* and *Goniotropis parca* first instar larvae. *Ozaena lemoulti*: (a) right antenna, dorsal view, arrow indicates the hyaline vesicle. (c) right maxilla, dorsal view, the box on the bottom shows a close-up of the obsolescent basal tooth. (e) mandibular retinaculum, dorsal view. *Goniotropis parca*: (b) right antenna, dorsal view, arrow indicates the hyaline vesicle. (d) right mandible, maxilla and labium, dorsal view, the box on the bottom shows a close-up of the well-developed basal tooth. (f) mandibular retinaculum, dorsal view.

**Fig 6:**
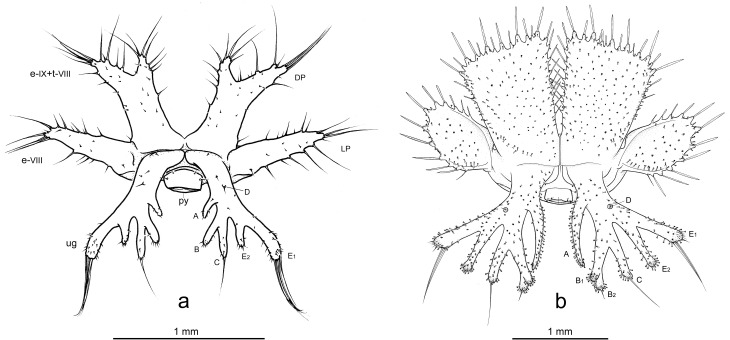
Illustrations of the terminal disks of *Ozaena lemoulti* and *Goniotropis kuntzeni* first instar larvae. (a) *Ozaena lemoulti*. (b) *Goniotropis kuntzeni* (modified from Moore & Di Giulio 2006). A-E_2_: notation of urogomphal lobes; DP: dorsal plate; e-VIII, e-IX: epipleurite VIII, IX; LP: lateral plate; py: pygidium; t-VIII: tergite VIII; ug: urogomphus.

**Fig 7:**
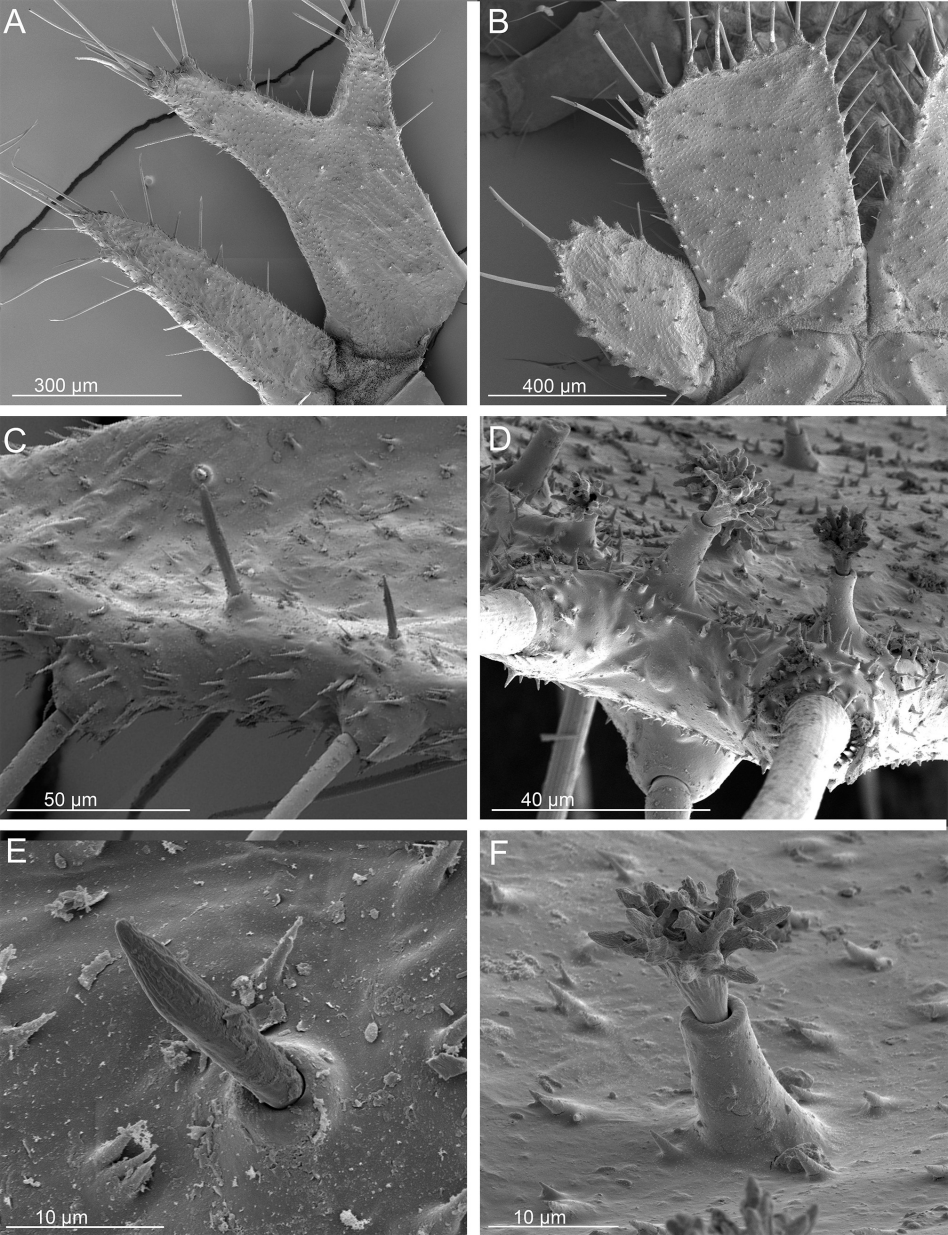
SEMs of the terminal disks of *Ozaena lemoulti* and *Goniotropis parca* first instar larvae. *Ozaena lemoulti*: (a) left dorsal and lateral plates. (c) sensilla S-I on dorsal plates. (e) close-up of sensillum S-I. *Goniotropis parca*: (b) left dorsal and lateral plates. (d) sensilla S-I on dorsal plates. (f) close-up of sensillum S-I.

**Fig 8:**
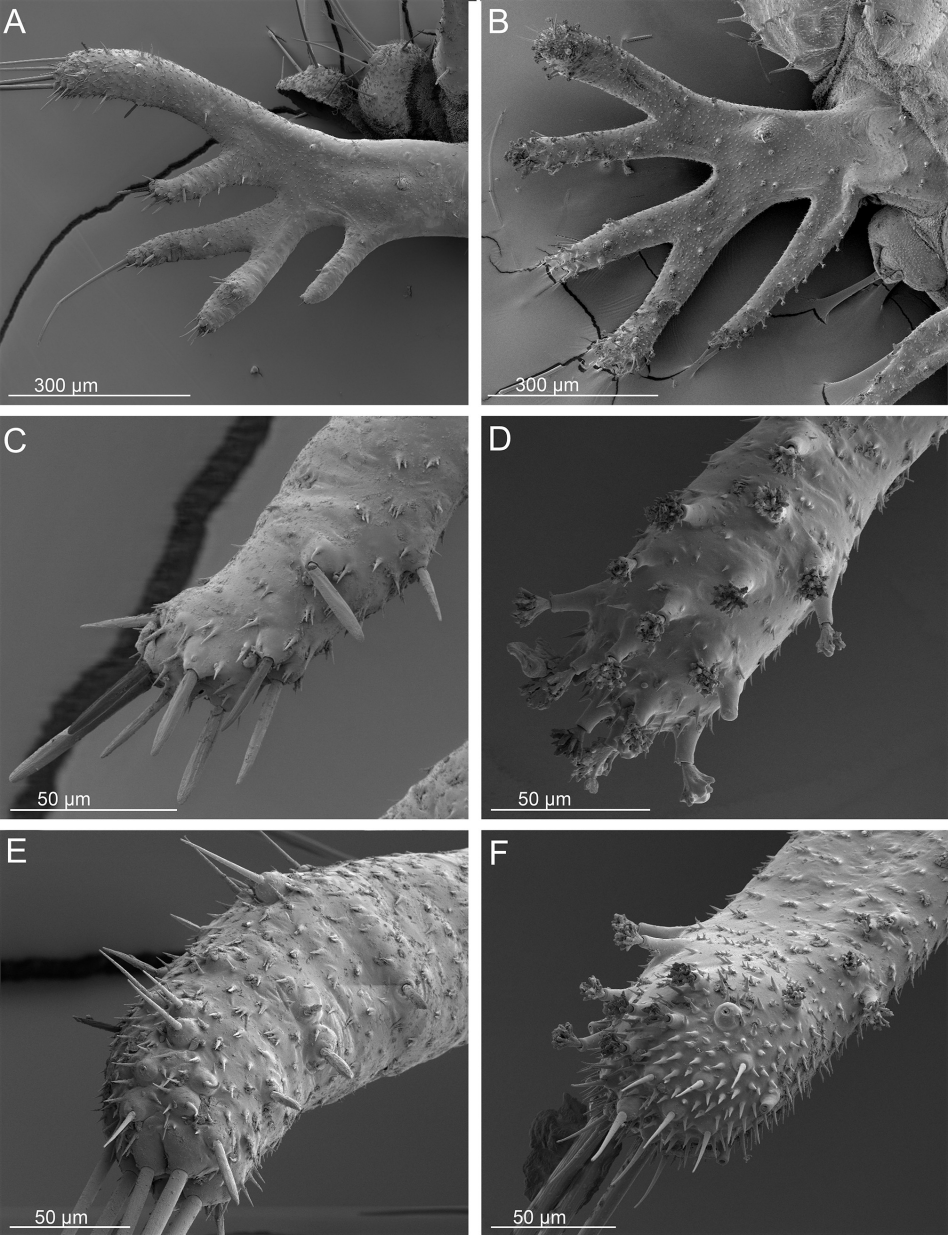
SEMs of the urogomphi of *Ozaena lemoulti* and *Goniotropis parca* first instar larvae. *Ozaena lemoulti*: (a) left urogomphus, dorsal view. (c) apex of E_2_ lobe. (e) apex E_1_ lobe. *Goniotropis parca*: (b) left urogomphus, dorsal view. (d) apex of E_2_ lobe. (f) apex E_1_ lobe.

Descriptions of the first instar larvae of *Ozaena lemoulti* and *Goniotropis parca* are provided in S1 Appendix and comparatively illustrated (Figs 3–8).

The main differences between the anatomy of the *O*. *lemoulti* larva and those of its close relatives and known burrow trappers, *G*. *parca* and *G*. *kuntzeni* are as follows.

1. The total body length, measured from tip of mandibles to apex of terminal disk, of *O*. *lemoulti* is 4.3 mm (Fig 3A and 3B), *G*. *parca* is 4.0 mm, and *G*. *kuntzeni* is 7.4 mm (Fig 3C and 3D).

2. The orientation of the head is prognathous in *Ozaena* but hyperprognathous in the *Goniotropis* species.

3. The *Ozaena* larva is completely blind, without stemmata (Figs 3A and 4A) whereas the larvae of *Goniotropis* have one stemma on each side of the parietale (Fig 3C).

4. The sensorial appendage on antennomere III is reduced, small and spherical in *Ozaena* (Fig 5A, arrow) but well-developed, elongate, conical, and apically pointed in *Goniotropis* spp. (Fig 5B, arrow).

5. The retinaculum of the mandible is slender, triangular, subapically directed in *Ozaena* (Fig 5E); but robust, hook-like, slightly curved inward in *Goniotropis* spp. (Fig 5D and 5F).

6. The lacinia is small, straight in *Ozaena* (Fig 5C, arrow) but well developed and hook-like in *Goniotropis* spp. (Fig 5D, arrow).

7. Galeomere II is short and slender in *Ozaena* (Fig 5C) but elongate and stout in *Goniotropis* spp. (Fig 5D).

8. Mesal stipital protuberance is obsolescent and positioned mesodorsally in *Ozaena* (Fig 5C, box), but it conspicuous, and positioned subbasally on inner side, and is directed forward in the *Goniotropis* spp. (Fig 5D, box).

9. The abdomen of Ozaena is extremely short and flat and is not contracted dorsally (Fig 3A and 3B); whereas the abdomen of the *Goniotropis* spp. is moderately short and flat, and contracted dorsally (upcurved) elevating terminal disk (Fig 3C and 3D).

10. The terminal disk of *Ozaena* is whitish, unsclerotized, and translucent (Fig 3A and 3B); but well sclerotized and yellowish in *Goniotropis* spp. (Fig 3C and 3D).

11. The terminal disk is much narrower (1.9 mm at level of lateral plates) in *Ozaena* (Fig 6A) than it is in the *Goniotropis* spp. 2.89 mm (Fig 6B).

12. The dorsal plates of the terminal disk are shorter in *Ozaena* (0.6 mm measured from base, near articulation, to medial apex) (Figs 6A and 7A); than in *Goniotropis* spp.(1.44 mm) (Figs 6B and 7B).

13. The shape of the dorsal plates of the terminal disk are different. In *Ozaena* they are thin, elongate, widely separated in the middle by a U-shaped space and apically two-lobed, the outer lobe bearing a brush of long setae (Figs 6A and 7A). In the *Goniotropis* spp. the dorsal plates are rectangular, slightly enlarged from base to apex and separated in middle by a thin, V-shaped space (Figs 6B and 7B).

14. The lateral plates of *Ozaena* area extremely thin and elongate, acutely triangular, more than 3 times wider than long (Figs 6A and 7A); in *Goniotropis* the lateral plates are suboval, auriform, and almost 2 times wider than long (Figs 6B and 7B).

15. The urogomphi are composed by 6 branches (Figs 6A and 8A) in *Ozaena* and 7 branches in *Goniotropis* spp. (Figs 6B and 8B). In *Ozaena* lobe A is the shortest, thin and highly reduced, about as long as E2, lobe B is simple, lobe C is much longer than E2 (Fig 8C), E1 is extremely long and thick, club-shaped and with a tuft of long setae at apex (Figs 6A, 8A and 8E). In *Goniotropis* spp. lobe A is slender and elongated, curved and tapered to tip, much longer than E2, lobe B is two-lobed at the apex (B1, B2), lobe C is as long as E2 (Fig 8D), lobe E1 and E2 are straight and subparallel sided, lobe D is small, dorsal, emerging from base of lobe E (Figs 6B, 8B and 8F).

16. The shape of sensilla S-I differs between the two genera. In *Ozaena* they are spiniform, and they are tightly inserted in slightly raised cuticular bases (Fig 7C and 7E); In *Goniotropis* spp. they are “rosette-like”, with short multilobed setae emerging from elongate, cylindrical cuticular bases (Fig 7D and 7F).

17. The shape of sensilla S-II differs between the two genera. In *Ozaena* they are thin and flexible, long, forming brushes at apices of dorsal and lateral plates, similar to cluster S-V on urogomphus lobe E1 (Figs 6A and 7A). In the *Goniotropis* spp. they are stick-like, hard, inflexible, lining the sides of dorsal and lateral plates (Figs 6B and 7B).

### Comparative anatomy of adults

**Fig 9:**
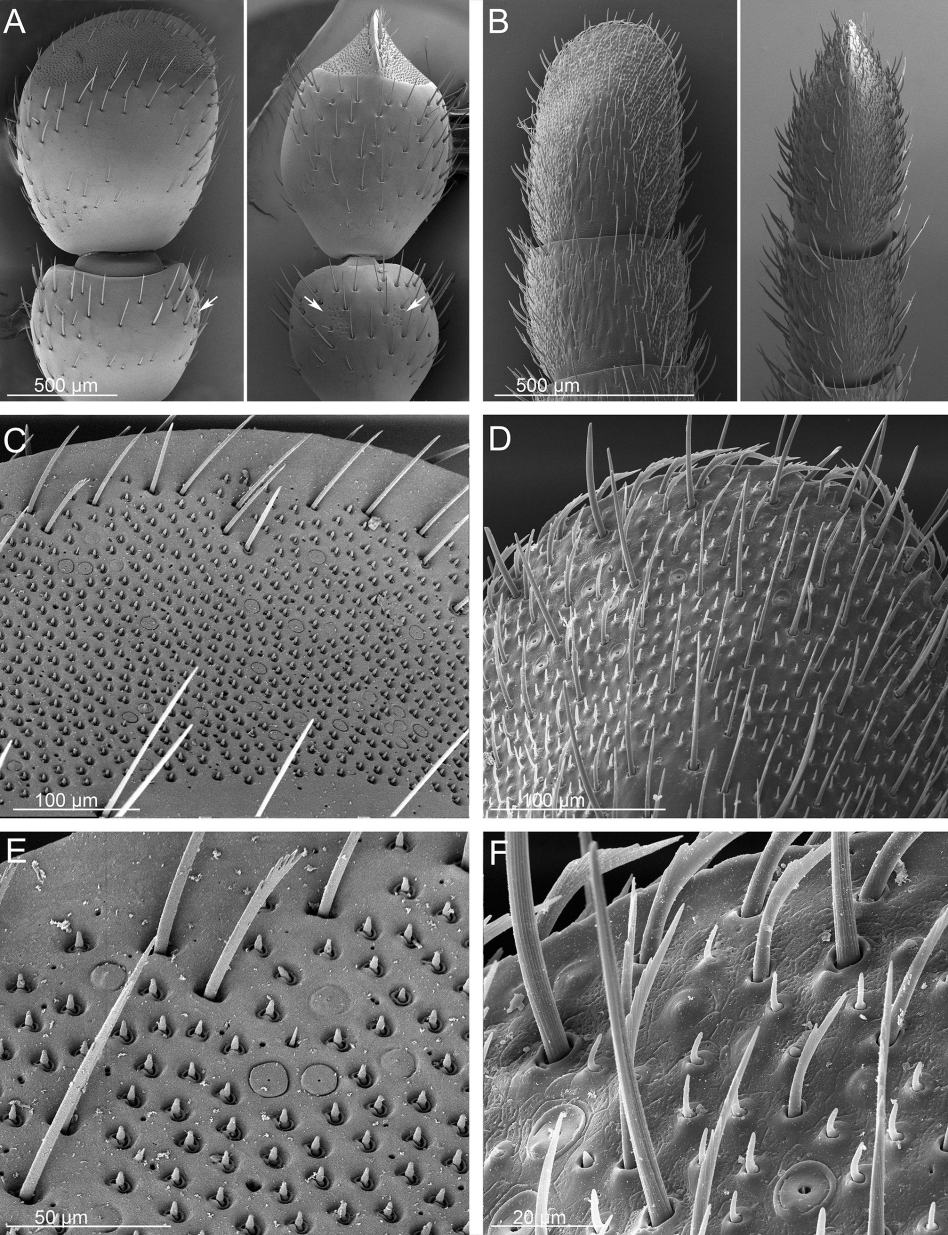
Antenna of *Ozaena lemoulti* and *Goniotropis parca* adults. *Ozaena lemoulti*: (a) apex of right antenna, dorsal view (left micrograph) and lateral view (right micrograph), arrows indicate the lateral sensory areas. (c) sensorial area on last flagellomere. (e) close-up of sensilla present on last flagellomere. *Goniotropis parca*: (b) apex of right antenna, dorsal view (left micrograph) and lateral view (right micrograph); (d) sensorial area on last flagellomere. (f) close-up of sensilla present on last flagellomere.

The moniliform antennae of *Ozaena* species (Fig 1E) are very different from those of all other ozaenines [30, 31], especially with respect to the shape of the last seven antennomeres (Fig 1E), which are distinctly globular and separated from one another by basal constrictions, giving this part of flagellum increased mobility. Moniliform antennae are typical of myrmecophilous beetles, including *Protopaussus* Gestro, and more distantly related Pselaphidae and Brentidae. The homologous antennomeres of *Goniotropis* species (Figs 1B, 1C and 9B) are dorsoventrally flattened and appressed. These flagellomeres are matte in *Goniotropis* spp. (Figs 1A,1B and 9B) and in most other ozaenines, but appear glossy in *O*. *lemoulti* (Figs 1E and 9A) due to the general loss of sensilla, especially sensilla basiconica, from most of the surfaces. In *Ozaena*, the apex of the last antennomere has a dense field of basiconic sensilla (Fig 9C and 9E), which are smaller than the curved and peg-like basiconic sensilla of other ozaenines. In this region, *Ozaena* also has some atypical coeloconic sensilla, with a relatively small medial pore, campaniform sensilla, short taste bristles and several apically fringed sensilla chaetica which surround the apical sensorial area (Fig 9C and 9E). The apical part of antennomere XI also has a distinct keel (Fig 9A, right micrograph), dividing the dorsal and ventral parts. The apical surface is covered with finely dotted microsculpture and many pores of different dimensions (Fig 9E), some of which produce filiform, paste-like material. Antennomeres V-X also have two small lateral sensorial areas, with two groups of sensilla basiconica (Fig 9A, arrows).

### Molecular identification of adult gut content

**Fig 10:**
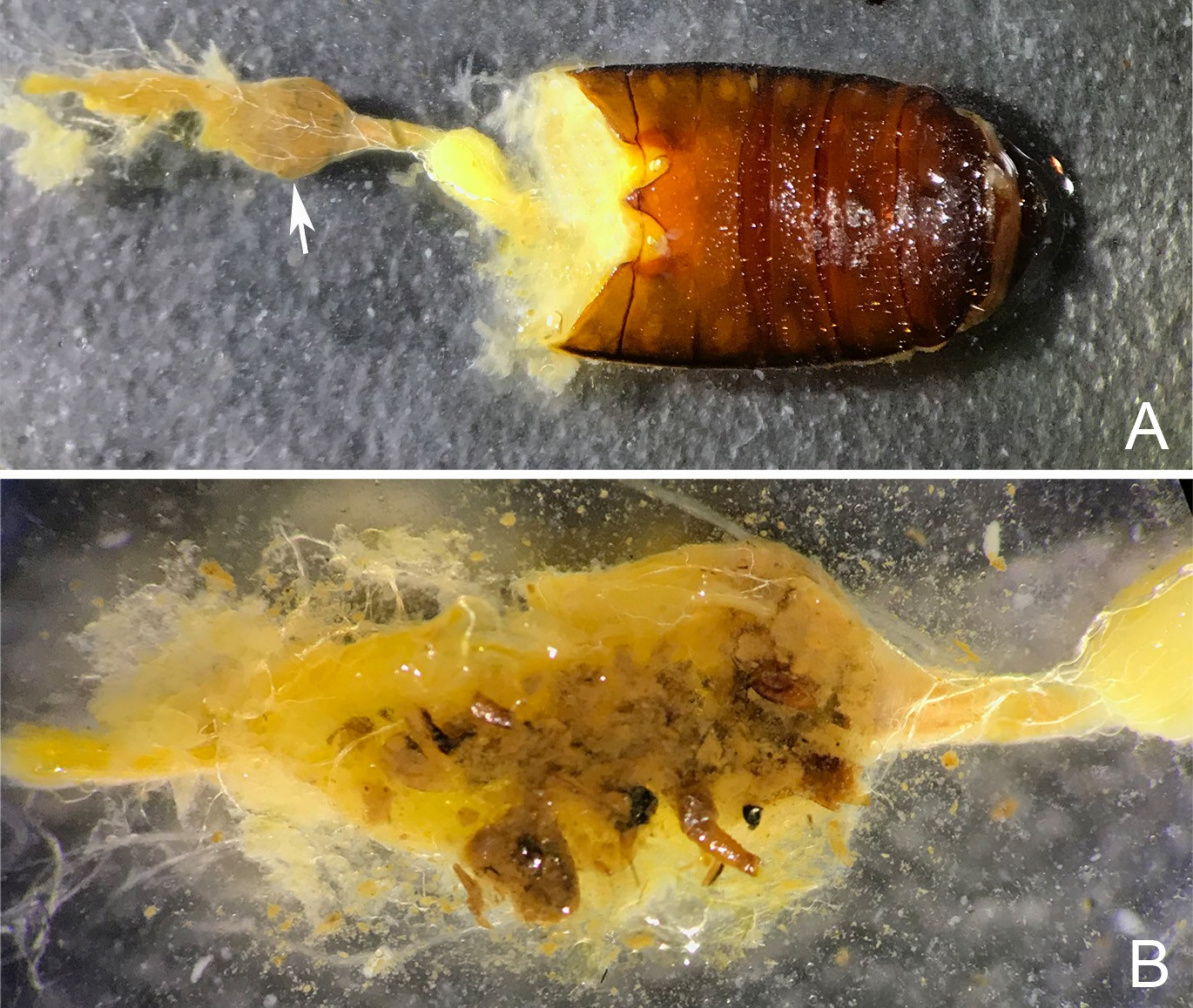
Dissections of *Goniotropis kuntzeni*. (a) dissected gut (ventral view) showing the crop (white arrow) and anterior portion of the midgut (yellow). (b) close up of the dissected crop with remnants of partially digested arthropods.

While the crops of *Goniotropis* adults were full of pieces of cuticle typical of other general arthropod predators (Fig 10A and 10B), the crops of *Ozaena* adults were full of a grayish pasty substance typical of other Paussinae species that feed by sucking the fluids from ant larvae, pupae and adults, including those in the myrmecophilous genus *Paussus* (WM and ADG personal observations).

Most of our attempts to amplify genes from the gut contents of adults, resulted in a successful amplification and clean sequence of the beetle itself or a sequence that was too messy to be meaningful. The successful results of our PCR survey of gut content are reported in Table 1. All sequences of the pasty substance in the gut of *Ozaena* spp. turned out to be from a *Camponotus* ant, including one 1116 basepair fragment of 28SrDNA from a specimen of *Ozaena lemoulti* from French Guiana. Individual fragments of the cuticular pieces from the guts of *Goniotropis kuntzeni* revealed that they feed on *Camponotus* as well as other arthropods in their environment (such as chrysomelid beetles).

**Table 1:** Results from sequence-based identification of gut contents.

Taxon	Locality	Gene (bp) (GenBank #)	Query cover	% similarity	Closest relative (GenBank #)
*Ozaena lemoulti* (DNA2530)	French Guiana	28S (1116) (MK253719)	100%	100%	*Camponotus* (JN134291.1)
*Ozaena martinezi* (DNA4763)	Bolivia	28S (330) (MK253720)	100%	99%	*Camponotus* (KX347429.1)
*Ozaena martinezi* (DNA4767)	Bolivia	28S (310) (MK253721)	81%	96%	*Camponotus* (KX347429.1)
*Goniotropis kuntzeni* (DNA4757)	Arizona (Walker Canyon)	COI (678) (MK253722)	99%	87%	*Camponotus* (JN134833.1)
*Goniotropis kuntzeni* (DNA4753)	Arizona (Walker Canyon)	COI (678) (MK253723)	100%	88%	*Chaetocnema* (KX943442.1)

## Discussion

Comparative anatomy of three life history stages reveals that *Ozaena* species have adopted a new, obligate nest parasite strategy for living with and exploiting ants as their sole source of food. While we do not have direct evidence of the *Ozaena* larva's behavior (we reared only one larva and preserved it before attempting to feed it), unique morphological modifications of the head and abdomen indicate that *Ozaena* larvae do not live in burrows but rather are free-living, mobile hunters. One might wonder what would motivate this shift. Burrow trapping is a highly specialized feeding strategy requiring muscle power, fine sensing capabilities, and motor skills. It is an effective way of feeding that simultaneously provides the soft larva protection from predation. For all other known ozaenine larvae, these benefits must outweigh any costs associated with this feeding strategy, such as energy needed to build and maintain the burrow and the risk of the burrow being disrupted, as the burrow trappers are very awkward and vulnerable when they are outside of their burrows. We hypothesize that the main motivation to leave the burrow is the opportunity that affords the larva to feed on non-mobile food, specifically ant brood, the most treasured, soft, protein rich, and fat rich resource in the nests.

Most eggs of adephagan beetles have a rather simple and thin chorion without a spongy air layer [42]. The presence of a thick air layer in Ozaenini and basal Paussini, classified as eggshell Type A, is considered as a synapomorphy of the Paussinae [32]. However, such an air layer has been secondarily and independently lost (eggshell Type B) or almost completely reduced (special deviation of Type A) in three lineages of myrmecophilous Paussinae: in the ozaenine genus *Physea*, living in leafcutter ant nests, in the basal paussine genus *Arthropterus* and in the “derivative paussids” (*sensu* Darlington [2]), for example of genera *Platyrhopalopsis* and *Paussus* [32]. For this reason, Kaupp et al. [32] predicted that within the Paussinae, there might be a connection between eggshell type and oviposition site and they considered there may be a correlation between Type B and myrmecophily. The presence of an air layer can be advantageous in terrestrial animals, since in arid conditions it can minimize the loss of water, while in humid conditions it prevents the respiratory membrane from being directly covered by a film of water. However, ant nests are very stable edaphic environments, constructed in ways that minimize the risks of drying, freezing or flooding. This is another reason that ant nests are so attractive to myrmecophiles. We speculate that under such stable conditions, a thick spongy air layer may easily be lost since it represents an energy cost no longer critical for embryo survival. We consider the absence of the spongy air layer in *Ozaena* as evidence that the egg develops inside ant nests.

Our hypothesis that the *Ozaena* eggs are laid inside their host ant nests, is further supported by the fact that the sensorial appendage on the third antennomere of first instar larva is greatly reduced. This olfactory structure is well-developed in the larvae of *Goniotropis*, as it is in all other mobile carabid larvae. In fact, it is especially well-developed in the planidia of parasitoid carabids, but it is reduced in later instars after they have found their host and become physogastric [43].

Comparative anatomy between *Ozaena* and its close relative *Goniotropis* (an effective burrow trapper) reveals that *Ozaena* larvae have abandoned the burrow to become highly mobile myrmecophiles. For example, *Ozaena* has a prognathous head, a unique trait within the tribe. All other ozaenine larvae have hyperprognathous heads, which allows them to grasp living insect prey with their mandibles during ambush burrow trapping. The *Ozaena* larva is completely blind, as are other known mymrecophiles within the Paussinae including *Eustra*, *Physea*, and Paussini. In addition, several mouthpart structures, that are used by other ozaenine larvae to dig their galleries and to grasp their prey tightly, are greatly reduced and modified, indicating that *Ozaena* larvae do not feed on struggling prey. For example, the mandibular retinaculum, lacinia, and basal tooth of the stipes is reduced, straight, and blunt in *Ozaena*, rather than being well-developed and hook-like as they are in their burrow- trapping relatives. Similarly, the robust spine-like setae (group gMX) that create a rasp on the dorsal surface of the stipes in the burrow trappers are soft, slender and elongate in the *Ozaena* larva.

Burrow trappers have shortened tergites relative to sternites creating a U-shaped body that allows its head to be close to the terminal disk door. The abdomen of *Ozaena* is highly modified resulting in a completely different overall body shape. The abdominal tergites and sternites are subequal in length, resulting in a flattened body shape rather than the U-shaped body shape typical of other ozaenine larvae. The terminal disk is not more sclerotized relative to other parts of the body (as it is in all known burrow trappers) and the components of the disk (urogomphi and lateral and dorsal plates) are elongated rather than flattened into wide plates that together form a rounded cover for their burrow openings. Further, the *Ozaena* larva also lacks modified sensillae along the edges of the terminal disk, which a burrow trapper uses to contact the edges of their burrow opening and seal it. *Ozaena* also lacks the short spiniform setae and setae with a frayed apex, indicating this larva does not use the terminal disk to sense prey presence. There are no specific structures that suggest *Ozaena* uses the terminal disk to interact with ants, such as are present among other known myrmecophiles such as *Physea* and *Paussus*.

All of these modifications indicate an adaptive shift from living in burrows and ambush hunting by burrow trapping (like in *Pachyteles* and *Goniotropis*) to a free-living mobile hunter. In fact, the *Ozaena* larva has the longest legs relative to body size of any known paussine larva.

Our hypothesis that *Ozaena* is a myrmecophile is bolstered by several aspects of adult anatomy. The adult females have many modifications of sensillae and presumably odorant binding proteins, to find their host ant nests, and they have sensitive ovipositors to lay their eggs in appropriate places. Adults have heavily sclerotized bodies to protect them from ant attacks, modified setae to help them spread the substances they produce that are attractive to the ants, and a cylindrical body shape to facilitate moving in the ant burrows (Fig 2). Indeed, our investigation of the physical state and molecular identification of gut contents, provides direct evidence that adult *Ozaena lemoulti* have adopted a liquid diet and specialize on sucking juices from *Camponotus ocreatus*.

*Camponotus* is a hyperdiverse genus, containing over 1000 species. While some species are known to nest underground, *Camponotus* species are commonly called carpenter ants because many establish their colonies in galleries excavated in damaged wood. This seems to be the case for *Camponotus ocreatus*, as we have observed both the ants and the ozaenines entering holes in the bark of Emory oaks. Many *Camponotus* species are known to be polydomous, having a primary nest with the queen and the brood and secondary satellite nests with other workers [44]. One intriguing possibility is that *Ozaena* larvae and adults have evolved the ability to take advantage of the rich resources of the primary nest.

Oftentimes larval anatomy is studied only minimally or it is completely unknown for entire lineages of holometabolous insects. This work demonstrates the value of studying the comparative morphology of both adult and larval anatomy in our efforts to understand the shifts in the natural history of both life stages which can greatly affect and even drive shifts in the evolution of entire insect lineages. Especially in myrmecophilous insects, the immatures are the most vulnerable life stage and must evolve structural, chemical or even acoustical strategies to survive and benefit from the valuable resources of the nest.

## Supporting information

S1 Appendix Descriptions of *Ozaena lemoulti* and *Goniotropis parca* first instar larvae. (PDF)
