# Supplementary material for: Out of the burrow and into the nest: Functional anatomy of three life history stages of Ozaena lemoulti (Coleoptera: Carabidae) reveals an obligate life with ants
Source: PLoS One. 2019 Jan 16;14(1):e0209790. doi: 10.1371/journal.pone.0209790 (PMC6334910; doi:10.1371/journal.pone.0209790)
Supplement: S1 Appendix — (PDF) [file pone.0209790.s001.pdf]

S1 Appendix. Descriptions of *Ozaena lemoulti* and *Goniotropis parca* first instar larvae.

Ozaena lemoulti first instar

Diagnosis.– Head prognathous (Fig 3A,B); abdomen flat, not upcurved (Fig 3A,B); terminal disk highly modified with dorsal plates thin, elongate, widely separated in the middle by a U-shaped space and apically two-lobed, the outer lobe bearing a brush of long setae (Fig 6A); lateral plates extremely thin and elongate, laterally projecting (Figs 6A, 7A); urogomphi with lobe A shortest, thin and highly reduced, about as long as E2 (Figs 6A, 8A); E1 extremely long and thick, club-shaped and with a tuft of long setae at apex (Figs 6A, 8A,E).

Measurements.– Body length about 4.3 mm (from tip of mandibles to apex of terminal disk); cephalic capsule maximum width (at base of antennae) 1.2 mm, medial length (from occipital foramen to anterior margin of frontoclypeolabrum) 0.82 mm; prothorax maximum width 1.1 mm, medial length 0.51 mm; terminal disk maximum width (at level of lateral plates) 1.9 mm; dorsal plates length (from base, near articulation, to medial apex) 0.6 mm.

Habitus and coloration.– Body soft, weakly sclerotized, not physogastric. Abdomen extremely short, bellows-like (Fig 3A,B), flattened (not contracted dorsally), subparallel sided (Fig 4C,D). Head, thorax and legs slightly sclerotized and pale yellow (Fig 3A,B); terebra, retinaculum and apex of mandibles, laciniae, anterior margin of frontal sclerite, labral spine, egg-bursters and claws thickly sclerotized and reddish brown to piceous (Fig 3A); abdomen and terminal disk whitish, unsclerotized, translucent (Fig 3A,B).

Microsculpture – Cephalic capsule, mouthparts, thoracic tergites and legs smooth, without or with only sparse, pointed microsculpture. Labral spine and anterior margin of epipharynx with spinulate microsculpture, resulting in a slightly serrate anterior margin (Fig 5D); lateral and anteromedial part of epipharynx with thin, elongate sculpticells medially directed; posteromedial part of epipharynx with many short rows of anteriorly directed spines; hypopharynx with many long concentric comb-like rows, each composed of hundreds of small anteriorly directed teeth; epi- and hypopharyngeal microsculpture, together with associated setae, forming a mouth filter apparatus; basal third of prementum with pointed microsculpture on dorsal surface. Membranous areas of body and sclerites of the abdomen rugulose to rugose, with pointed or multi-pointed sculpticells, sparse near setae. Surface of plates and urogomphi of terminal disk (Figs 7C,E; 8C,E), as well as pygidium, with pointed to multipointed microsculpture.

Chaetotaxy - Frontoclypeolabrum (Fig 4A) without additional setae; FR1, FR2 and FR3 subequal in length; FR1 and FR3 at anterior third of egg bursters; FR2 at basal third, and lateral to egg bursters; FR4* slightly shorter than FR3, anteromedial to frontal area; seta FR5 absent, possibly replaced by a pore; FR6 small, slightly lateral and posterior to FR7; seta FR7 longer than other frontal setae, almost twice as long as FR1; setae FR8 and FR9 moderately long, medially directed, inserted ventrally at anterior corners of median prominence; setae FR10* and FR11* just behind transverse frontal keel; seta FR 11* slightly posterior to and longer than FR10*; labral spine with 2 minute anteriorly directed setae on dorsal surface. Parietale (Fig 4A-B) with some minute additional setae between PA8* and basal stem of epicranial suture; 3 small ventrolateral additional setae posterior to PA14; PA8* elongate, slightly anterior to PA9*. Antennomere I (Figs 4A-B, 5A) with 5 additional setae: 2 short dorsolateral setae on basal half, and a crown of 3 longer setae subapically; II without additional setae; III with seta AN1 displaced anteriorly; IV with AN6 short. Mandible (Figs 4A-B, 5E) with one additional lateral seta just more distal to retinaculum; seta MN1 short; 5 additional pores on dorsal surface. Setal group gMX (Fig 5C) on stipes composed of approximately 10 elongate and thin setae distributed especially along occlusal margin; seta MX5* and another subapical additional seta at the base of maxillary palpi spine-like; seta MX6 small, subapical on lacinia; MXd* ventral at apex of galeomere I; seta MX8* subapical, close to MX9*; small sensorial area (composed of 3 short lateral and 1 longer medial basiconic sensilla) present at apex of galeomere II (Fig 5C); maxillary palpomere IV with small dorsolateral additional seta on medial side, 1 ventral pore and 3 longitudinal subapical digitiform sensilla and apical sensorial area composed of several peg-like sensilla. Prementum (Figs 4A-B) with about 26 additional spine-like setae (13 on each side) on lateral and dorsal surfaces, inserted in protuberances; seta LA1 close to midline; labial palpomere II with 2 additional setae, 1 mesodorsal and 1 small ventrolateral, 3 longitudinal subapical digitiform sensilla and apical sensorial area composed of several peg-like sensilla. Each side of epipharynx with 3 small additional setae and 1 pore posterior to couple FR8-FR9. Pro-, meso- and metanotum (Figs 2A, 4A) with 2 or 3 additional setae and several pores on each side of ecdysial line; seta PR14* elongate on discal area. Trochanter (Fig 3B) with many elongate setae present mostly subapically on ventral side, about as long as TR4; TR8 shorter and thinner than TR4. Prosternum with gPS group composed by a curved line of 8 small setae; meso- and metasternum with 3 small additional setae on each side, and MS4* extremely long. Abdominal tergites I–VII (Figs 2A, 4C) with reduced setation. Tergal side of dorsal and lateral

plates of terminal disk (Figs 2A, 4C) with stiff pointed setae (sensilla S-VII) of various sizes, with cylindrical bases protruding from plates: about 10 on each dorsal plate (epipleurite IX + tergite VIII) and about 6 on each lateral plate (epipleurite VIII); margins of dorsal and lateral plates (Figs 6A; 7A,C) with sensilla S-II long, thin and flexible, forming brushes at apices of dorsal and lateral plates; each dorsal plate with about 15 S-II, irregularly disposed along distal and medial margin; margin of each lateral plate with 13 sensilla S-II, those at apex of lateral plates more elongate; ventral side of terminal disk with a few, sparsely distributed, short (10-30 μm) spiniform sensilla, perpendicular to the surface, with seta irregularly wrinkled and blunted at tip, tightly inserted in slightly raised cuticular bases; because of their position and orientation, their homology with sensilla S-I is very likely (Fig 7A,C,E): about 10 S-I on each dorsal plate, mostly basally and medially, and about 6 on each lateral plate. Epipleurites and hypopleurites of abdominal segments I–VIII with several setae and elongate sensilla S-II (see Figs 2A-B; 4C,D). Urogomphi (Fig 8A) with several, sparse S-I, mainly on dorsal and apical areas of branches (Fig 8C,E); branch A with 4-5 short spiniform sensilla dorsally; B with 10 short spiniform sensilla of different sizes apically and 3 subapically, C at apex with 1 long S-II and 6 short spiniform sensilla, especially concentrated on ventral side; E2 with 10-12 short spiniform sensilla; E1 with several (about 30) short spiniform sensilla subapically, cluster S-V and 2 long S-II apically (5 long setae in total); branch D with 1 S-III apically. Pygidium with 2 lateral spiniform sensilla.

Head.– Cephalic capsule (Fig 4A-B) distinctly transverse (width/length ratio = 1.46), prognathous, broadly rounded laterally, regularly tapered at basal half into a distinct neck; maximum width at antennal insertions about twice as wide as at occipital foramen, occipital foramen posterodorsal; tentorium with posteromedial processes joined forming a subrectangular bridge (tentorial bridge). Frontoclypeolabrum (Fig 4A) shield-like, distinctly transverse (width/length ratio = 1.4), with surface medially convex and laterally concave; basal stem of epicranial suture moderately short, anterior frontal arms distinctly sinuate; egg-bursters (Figs 2A, 4A) keel-like, composed of two longitudinal, multispinulate carinae, each consisting of about 50 apically directed teeth, anteriorly preceded by a larger triangular spine; carinae parallel, almost half frontoclypeolabrum length, widely separated, placed between FR1 and FR3. Frontoclypeolabrum (Figs 4A) with two anterior margins: dorsal margin smooth, moderately curved and anteriorly prominent, forming a transverse keel (see Di Giulio et al. 2003 and Di Giulio and Moore 2004 for a discussion on homology) extended to adnasalia, ventral margin slightly serrate, medially produced into a subrectangular labral tooth (labral spine *sensu* Beutel

1992) partially 2-lobed apically; adnasalia slightly rounded, not protruding. Parietale (Figs 2A; 4A) without stemmata; ocular and cervical grooves absent; ventral walls of parietale medially fused forming a complete single gular suture (Figs 2B; 4B). Antennae (Figs 4A; 5A) 4-jointed, slightly shorter than mandibles, forwardly directed; antennomere I about 2 times longer than II and about 1.5 times longer than III; IV slightly shorter than III; III with small, globular sensorial appendage (Fig 5A, arrow). Mandibles (Figs 4A-B, 5C) long and slender, falcate, sharp at apex, about 2.5 times as long as their basal width, evenly curved along lateral margin; penicillus absent; terebra double-edged: dorsal cutting edge slightly convex beyond retinaculum, then concave near apex; ventral cutting edge regularly concave; retinaculum subtriangular, with pointed apex, subapically directed. Maxilla (Figs 4A-B, 5C) with undivided cardo, long and slender stipes, 4-jointed palp, 2-jointed galea and 1-jointed lacinia; stipes only slightly curved inward, about 3 times as long as their basal width; mesal stipital protuberance obsolescent (Fig 5C, close-up window), present subbasally on inner side. Setal notches absent along inner side; maxillary palpomeres gradually decreasing in length from I to III; IV digitiform, distinctly longer than others, slightly longer than II and III combined. Galeomere I short; II cylindrical, decreasing in size from base to apex, about 2 times as long as I, slightly curved inward at distal half. Lacinia short and straight, blunt tipped, basally fused with stipes (Fig 5C). Labium (Fig 4A-B) with sclerotized prementum and 2-jointed palps; prementum distinctly transverse with setal notches present on dorsal surface and sides; ligula elongate and subrectangular, slightly shorter than labial palpomere I; labial palpomere I slightly wider than II; II digitiform, about 2 times as long as I.

Thorax.– Tergites of pro- meso- and metanotum very similar (Fig 3A), transverse, about two times wider than long; pronotum slightly more suboval than subsequent tergites (Fig 4A). Longitudinal ecdysial line well marked on pronotum, but present also on meso- and metanotum.

Spiracles.– Thoracic and abdominal spiracles (Fig 4A,C) annular with conical, protruding peritremes. Mesothoracic spiracles anterolaterally positioned on mesopleura, diameter almost 2 times wider than diameter of abdominal spiracle I. Abdominal spiracle I diameter slightly wider than diameter of abdominal spiracles II–VII, II–VII subequal, VIII extremely small, placed basally between insertion of dorsal and lateral plates of terminal disk (Fig 4C).

Legs.– Legs long and stout, 5-jointed (Fig 3A-B), progressively longer from fore- to hindleg. Coxa cylindrical, elongate, about as long as trochanter and femur combined; trochanter obliquely truncate and ventrally expanded at apex, about as long as femur and tibia combined; femur about

twice as long as tibia, slightly increasing in size from base to apex; tibia cylindrical, about as long as tarsus; tarsus more slender than tibia, conical, tapered from base to apex, with 2 sharp claws; anterior claw long, regularly curved from base to apex; posterior claw shorter, thinner and almost straight.

Abdomen.– Abdominal segments I–VII very similar (Figs 2A-B, 4C-D), bellows-like, flattened (not contracted dorsally), subparallel sided. Abdominal sclerite boundaries barely discernable, recognized by reduction of pointed microsculpture, reduction of protrusions, and presence of setae or sensilla S-II; segments not widening from I to VII. Each segment dorsally flattened, with slightly swollen, setiferous pleural and sternal areas. Hypopleurites I-VII similar. Epipleurites distinctly protruding, similar in size from I to VII; epipleurites VIII (Figs 4C-D, 6A) flattened and enlarged into 2 sclerotized lateral plates, about as long as dorsal plates; lateral plates extremely thin and elongate, sbtriangular, laterally projecting, almost 3 times wider than long; epipleurites IX greatly enlarged and fused with tergum of segment VIII into a terminal disk; terminal disk (Figs 6A, 7A) completely divided into 2 rectangular plates (dorsal plates), widely separated by a U-shaped space; each dorsal plate slender, elongate, subparallel sided, distinctly enlarged and two-lobed; lateral lobes triangular, lateral lobe almost double than medial lobe and laterally projecting (Figs 6A, 7A); lateral plates, dorsal plates and urogomphi articulated at base by membranes, dorsal and lateral plates folded against the urogomphi in resting position. Median, inner and outer sternites not apparent. Urogomphi (Figs 6A, 8A) wide, branched, each composed of 6 lobes: A, B, C, D, E2, E1 (respectively from inner to outer) mostly developed on a single plane; A shortest, inserted more basally than others, digitiform, thin, almost straight, posteromedially projecting, about as long as lobe E2; B short, with remnants of a former subapical subdivision in two B1 (dorsal) and B2 (ventral); B1 almost completely absent, represented by a small dorsal, subapical, setipherous protuberance; C slightly longer and more slender than B; E2 small and thin, slightly bent laterally; E1 club-shaped, extremely long and thick; lobe D obsolescent, dorsal, emerging perpendicularly to plane of other branches (Figs 6A, 8A), slightly more proximal than level of A; pygidium (Fig 6A) flattened, medioventral between urogomphal insertions.

***Goniotropis parca* first instar**

The larva of *G. parca* (Figs 7B,D,F; 8B,D,F) is very similar to that of *G. kuntzeni* (see Moore

and Di Giulio 2006), both in shape and setation. For an extensive description and illustration of the egg (Fig 2B,D,F) and of the main larval characters (Fig 3C-D, 6B) and setation of *Goniotropis kuntzeni* we refer to Moore & Di Giulio (2006). Here, we highlight only the main differences between *G. parca* and *G. kuntzeni*.

Measurements – Body length about 4 mm (from tip of mandibles to apex of terminal disk); cephalic capsule maximum width (at base of antennae) 1 mm, medial length (from occipital foramen to anterior margin of frontoclypeolabrum) 0.62 mm; prothorax maximum width 0.87 mm, medial length 0.5 mm; terminal disk maximum width (at level of lateral plates) 1.5 mm; dorsal plates length (from base, near articulation, to medial apex) 0.68 mm.

As compared with *G. kuntzeni*, *G. parca* is relatively smaller and more compact; the cephalic capsule is shorter, more rounded and transverse; stemmata are relatively smaller; antennae with shorter and broader antennomeres, and slightly bigger hyaline vesicle; mandibles are shorter and stouter, with retinaculum less curved inward; maxillae with stipes shorter, broader and less curved, and shorter last palpomeres; last labial palpomeres shorter; legs with segments and claws distinctly shorter; terminal disk with dorsal plates more rectangular and subparallel-sided, only slightly broadened from base to apex, and more widely separated in the middle; lateral plates more suboval and less laterally projecting; urogomphi with all branches shorter and stouter, with fewer S-I sensilla.

References

Di Giulio A, Fattorini S, Kaupp A, Vigna Taglianti A, Nagel P. Review of competing hypotheses of phylogenetic relationships of Paussinae (Coleoptera: Carabidae) based on larval characters. Syst Entomol 2003;28: 509-537.

Di Giulio A, Moore W. The first-instar larva of the genus *Arthropterus* (Coleoptera: Carabidae: Paussinae): implications for the evolution of myrmecophily and phylogenetic relationships within the subfamily. Invertebr Syst 2004;18: 101-115.

Moore W, Di Giulio A. Description and behaviour of *Goniotropis kuntzeni* larvae (Coleoptera: Carabidae: Paussinae: Ozaenini) and a key to genera of Paussinae larvae. Zootaxa 2006;1111: 1-19.
